# Leveraging the Web and Social Media to Promote Access to Care Among Suicidal Individuals

**DOI:** 10.3389/fpsyg.2018.01338

**Published:** 2018-08-14

**Authors:** Charles-Edouard Notredame, Pierre Grandgenèvre, Nathalie Pauwels, Margot Morgiève, Marielle Wathelet, Guillaume Vaiva, Monique Séguin

**Affiliations:** ^1^Department of Psychiatry, Centre Hospitalier Regional et Universitaire de Lille, Lille, France; ^2^SCALab, Centre National de la Recherche Scientifique, Lille, France; ^3^McGill Group for Suicide Studies, Douglas Institute, McGill University, Montreal, QC, Canada; ^4^Fédération Régionale de Recherche en Psychiatrie et Santé Mentale des Hauts-de-France, Lille, France; ^5^Department of Psychiatry, Fondation FondaMental, Hospital Albert Chenevier, Créteil, France; ^6^Department of Public Health, Centre Hospitalier Regional et Universitaire de Lille, Lille, France; ^7^Department of Psychology, Université du Québec en Outaouais, Gatineau, QC, Canada

**Keywords:** suicide, prevention, Internet, online systems, social media, access to care, help seeking

## Abstract

After two decades of exponential development, the Internet has become an inseparable component of suicide prevention matters. More specifically, social media has turned out to be a privileged space for suicidal individuals to express their distress and seek support. Although this tendency carries with it specific risks and challenges, it creates unprecedented opportunities to face the challenges of help seeking and access to care. In this paper, we present the empirical, technological, and theoretical evidence supporting the implementation of a digitally augmented prevention policy that would increase its reach. Congruent to the clinical observations and theories on the help-seeking process, we argue that social media can help undertake three main functions of increasing proactivity to bring suffering Web users to care. The gateway function relates to the properties of social media interactions to leverage help-seeking barriers and enable ambivalent individuals to access the mental healthcare system. The communication outreach function aims to broadcast pro-help-seeking messages, while drawing on the functional structure of the social media network to increase its audience. The intervention outreach function consists in using machine learning algorithms to detect social media users with the highest risk of suicidal behaviors and give them a chance to overcome their dysfunctional reluctance to access help. We propose to combine these three functions into a single coherent operational model. This would involve the joint actions of a communication and intervention team on social networks, working in close collaboration with conventional mental health professionals, emergency service, and community resources.

## Introduction

With a number of users that has exploded from half a million in 2000 to almost 4 billion in 2017 (Internet Live Stats, 2016), the advent of the Internet represents one of the most dramatic social and technological evolution of the last two decades. It has deeply affected the way we interact, communicate, and access information. With respect to suicide, literature increasingly acknowledges the new or compounded threats but also original prevention opportunities that the Web has brought out (Durkee et al., 2011; Daine et al., 2013; Robert et al., 2015; Krysinska et al., 2017). This ambivalence was qualified as a “double-edged sword” (Tam et al., 2007; Robert et al., 2015). On the one hand, the Internet gave easy and immediate access to prevention information and help resources, fostering the development of peer-support communities. On the other hand, it gave pro-suicide websites visibility, paved the way for cyber-bullying, and increased dissemination of high suicide contagion risk content. The latest Web generation opened more optimistic perspectives for suicide prevention. The meta-restructuration, technological advances, and collaborative innovations it implies (Aghaei, 2012) represent a unique opportunity for prevention strategies not only to keep pace with the digital evolutions (Tam et al., 2007) but also to exceed them.

One of the most relevant prevention issues that the Web may help to address concerns access to care. Worldwide, 800,000 people still die by suicide each year (World Health Organization, 2014) and 3–16% of the population have had suicidal thoughts at least once (Nock et al., 2008). Research indicates that only 24% of suicidal individuals accessed mental healthcare in the month prior to their death (Ahmedani et al., 2014). While access to appropriate care is recognized as a crucial component of suicide prevention (Tondo et al., 2006; Campo, 2009; World Health Organization, 2014), barriers to help seeking appear to be stronger for vulnerable populations (Pirkis et al., 2003; Farand et al., 2004; Wu et al., 2010) and for those suffering from severe suicidal ideations and depressive symptoms (Reynders et al., 2015).

A growing number of authors have proposed using the Web and social media to overcome help-seeking barriers among suicidal individuals (Chan et al., 2017). Reasons why people who are suffering may decide against formal assistance can be categorized into four non-exclusive classes: (1) stigma barriers, in the form of public, perceived, or self-stigmatizing attitudes toward suicide, mental illness and mental healthcare, which often generate guilt, shame, self-blame, or inhibition; (2) structural barriers, such as accessibility, cost, visibility, or inconvenience issues; (3) psychological barriers, including lack of emotional competence, poor emotional expression, or excessive self-reliance; and (4) beliefs about care providers, encompassing concerns about confidentiality, trustworthiness, or competence (De Leo et al., 2005; Gulliver et al., 2010; Barker et al., 2011; Niederkrotenthaler et al., 2014). Social media promotes expression and interactions under the principles of anonymity and freedom of speech, and suicidal statements are frequent on the Web (Ruder et al., 2011; Sueki, 2015). Posting suicidal warnings on the Web could thus be interpreted as a more accessible, bearable, or affordable alternative to face-to-face help seeking (Gould et al., 2002; Michelmore and Hindley, 2012).

In this paper, we argue that public health could use social media advantageously to increase modern prevention policies. We propose a non-exhaustive description of available technological tools, epidemiological evidence, and theoretical concepts that could be relevant as a foundation for a Web-based strategy to promote and facilitate access to care for suicidal individuals. First, we describe the main functions that such a strategy could undertake, namely working as a gateway to healthcare, promoting help seeking via outreach communication and pro-actively bringing help *to* at-risk Web users via outreach intervention. Then, we propose a synergic integration of these three components in an operational, digitally augmented public health model.

## The Web as a Gateway to Formal Mental Healthcare

According to Rickwood et al. (2005), help seeking can be modeled as an active process requiring four sequential steps: awareness of the problem, expression of need for help, availability of support, and willingness to seek out help. These steps are distributed along a gradient of increasing motivation to action, which spans from personal contemplation to interpersonal solicitation. The four above-mentioned categories of help-seeking barriers specifically relate to different steps of this model. For instance, while psychological barriers reduce de propensity to realize and express emotional disturbances, structural barriers hinder the factual possibility to access healthcare. Stigma and beliefs about care provision negatively alter the approach/avoidance motivational balance for acting out (Rickwood et al., 2005; Gulliver et al., 2010; Luxton et al., 2011).

The various proposals to make the Internet a stepping stone to formal health are consistent with the sociological models that assume that help-seeking behaviors are mostly driven by interpersonal systemic determinants (Chan et al., 2017). The Gateway Provider Model, in particular, predicts that involving intermediary actors who know the community resources, interact with people who need help and refer them to appropriate services would enhance general access to care (Stiffman et al., 2004). Several properties of the Web suggest that online prevention interventions could endorse the role of gateway to reduce both structural and personal barriers to help. (1) Accessibility from almost any private or public place helps to skirt the constraints of distance from local services (Robert et al., 2015; Chan et al., 2017). It also facilitates discretion, which can alleviate interpersonal inhibitions to seek help. (2) Affordability compensates the structural barrier of cost and eases access to care for financially dependent individuals. (3) Timeliness guarantees access to support including outside work hours. It allows for attunement with the short timescale within which both severity of suicide ideation and motivation to ask for help can fluctuate (Miller, 2009; Luxton et al., 2011). (4) Anonymity and privacy are supposed to make the interaction less confrontational (Miller, 2009) and foster expression and self-disclosure (Chester and Glass, 2006; Krysinska and De Leo, 2007; Robert et al., 2015). (5) Impression of control comes with anonymity. It can secure individuals who cannot afford strong interpersonal commitment by giving them the opportunity to exit the conversation at any point (Robert et al., 2015).

This facilitation role, however, has its downside. Anonymity, in particular, severely hampers the possibilities of emergency interventions in case of imminent suicide risk. In addition, low interpersonal commitment gives the therapeutic link a labile dimension, with an increased risk of losing connection with the person. The Web could thus be placed at the extremity of a continuum ranging from high access probability but limited therapeutic engagement and possibilities of actions, to lower spontaneous contact probability but greater interpersonal commitment and larger scope of interventions. Under this perspective, the gateway function of Web-based interventions should not be regarded just as an entryway but also as a way to engage and reinforce the patient’s therapeutic commitment.

## Communication Outreach

As a first degree of proactivity, communication outreach consists in drawing on communication strategies to encourage suicidal individuals to use prevention services. Social media has opened new perspectives to the promotion of access to care. Beyond the simple setting up of passive websites, it offers to increase the outreach of prevention information. Literature has addressed two important related issues to inform communication strategies.

The first issue concerns the type of content that should be broadcasted to increase the probability that suicidal Web recipients will seek help. Indications come from “media effect studies” that have examined the impact of disseminating information on suicidal behaviors. In particular, Niederkrotenthaler et al. (2010) found that suicide stories can be linked to a significant decrease in suicide rates, provided that they respect some characteristics. The so-called “Papageno effect” has been described for reports of individuals who overcame a suicide crisis, but also suggested for similar fictional stories (Till et al., 2013; Notredame et al., 2017). Since numerous digital modules facilitate the broadcast of crisis-mastery stories to which suicidal individuals can identify (Luxton et al., 2012), spontaneous testimonials from celebrities or adolescents have flourished on social media (see, for example, Daoust, 2017; iamjordyndunlap, 2017). In terms of public health policies, the question is now about how to harness the collaborative nature and outreach potential of social networks to maximize the potential of preventive communication (Luxton et al., 2011). This would mean stimulating the production of such contents by both prevention stakeholders and lay Web users, but also structuring the creation process in an evidenced-based perspective. Several prevention organizations, such as Samaritans, have produced their own video storytelling depicting suicide survivors (Samaritans, 2017). In the same vein, Thiha et al. (2016) proposed to boost a school-based suicide prevention program with an interactive Web interface designed to help peer leaders make personal stories valuable contents to promote access to care.

The second issue has been raised by the applications of the network sciences to the field of suicidology. Two recent studies explored the architectural and dynamic properties of the networks formed by microbloggers who posted suicidal comments (Fu et al., 2013; Colombo et al., 2016) and led to similar observations. (1) The networks formed by suicide-posting microbloggers are characterized by a low density (i.e., low number of effective connections regarding the virtual number of possible connections), but a high degree of interconnectivity (i.e., high number of reciprocal or triangle connections). While sparsity is supposed to increase the speed of dissemination of the information, the strong reciprocity between posters of suicidal messages suggests potential recursion of the information, thus augmenting the impact of the messages. (2) Some microbloggers play a key role in the networks. On the one hand, influential posters (*influencers)* are those microbloggers who have a lot of followers but few friends, and whose posts are widely spread over the network. On the other hand, *influential reposters (linkers)* have both a significant number of friends and followers, such that they bridge the small disconnected hubs of which the network is composed.

Although deserving replications, lessons could be drawn from these observations to build outreach communication strategies. The structural and functional properties of social media allow for fast dissemination of messages that may be received redundantly by users. This communication strength is versatile. On the one hand, it reinforces the reach and impact of suicide contents, thus majoring the risk of contagion. On the other hand, it could be an exceptional channel to broadcast and boost the influential potential of pro-help seeking messages; however, the optimization of a communication strategy on social media must be tightly tailored to the structure and function of the targeted network. For instance, involving *influencers* may be a relevant way to ensure the broadest dissemination of Papageno messages. *Linkers* could also be usefully recruited as gatekeepers because of their privileged position to detect and signal worrisome messages.

## Outreach Intervention

A significant proportion of suffering Web users may remain impervious to the gateway or communication outreach strategies, although clearly needing support. The only way to compensate for this strong “help-negation” (Rudd et al., 1995) would thus be to bring help directly to them and try to reduce the barriers to formal help seeking *in situ*.

For practical and ethical reasons, proactivity should remain proportionate to the actual risk level. Estimation of the probability that Web users will engage in suicidal behaviors is an essential perquisite for any intervention outreach. In the field of suicidology, several authors have proposed to use artificial intelligence to automatically segregate high-risk messages from the mass of potentially concerning posts published on Twitter^®^ (Abboute et al., 2014; Homan et al., 2014; Burnap et al., 2015), Weibo^®^ (Lv et al., 2015), or Facebook^®^ (Thompson et al., 2014). The process includes four steps: (1) social media data are mined to extract the messages that contain one of a list of warning key words or expressions; (2) detected posts are decomposed in quantifiable functional linguistic features; (3) a statistical classifier is trained on a sample of pre-labeled posts to distinguish between high and low-risk messages (Thompson et al., 2014; Moulahi et al., 2017); and (4) the performance of the trained machine is tested against human coders. Depending on the feature sets and the nature of the classifier, authors have found precision rates (i.e., proportion of truly high-risk messages among those classified as such by the algorithm) ranging between 0.6 and 0.8% (Abboute et al., 2014; Homan et al., 2014; Burnap et al., 2015, 2017; O’Dea et al., 2015) and recall rates (i.e., number of truly high-risk messages picked out by the algorithm among the total number of high-risk messages in the sample) between 0.65 and 0.75 (Homan et al., 2014; Burnap et al., 2015, 2017; O’Dea et al., 2015).

The detection performance that has been reached so far led some authors to argue for implementation as a first step in an online outreach process (Robert et al., 2015; Chan et al., 2017). Literature offers numerous promising examples of Web-based interventions to prevent suicide (Barak, 2007; King et al., 2015; Robinson et al., 2015, 2016b; Mokkenstorm et al., 2017). The goal is either to extend the conventional care system with online psychotherapy or support groups or to serve as an entryway to this system. An example is the Israeli SAHAR program, which consists of an online platform that enables distressed Web users to have synchronous or asynchronous online interactions with helpers (Barak, 2007). With a further degree of proactivity, the American Foundation for Suicide Prevention developed organized systematic screenings of US college students via self-administered online questionnaires. All students were able interact with a counselor via a secured website. In addition, high-risk students were reached by e-mail and urged to attend in-person evaluation and treatment (Haas et al., 2008).

Nevertheless, to the best of our knowledge, no author has proposed online gateway intervention on an ecological outreach basis, i.e., for individuals who did not spontaneously ask for help, nor participated to formal screening procedures. Outreach intervention adds two important operational challenges to those of more traditional Web interventions. (1) Efficient and respectful messages, typically focusing on personalization, solicitude, or sense of belonging (Whiteside et al., 2014) would have to be designed to start the conversation with potentially unwilling individuals. (2) Formal clinical techniques would have to be developed to progressively increase the commitment of Web users who are not convinced *a priori*. There is no standard for online counseling (Miller, 2009). Nevertheless, outreach interventions should integrate several recognized characteristics of Web communication. For instance, textual interactions give no access to para-verbal cues but open to new possibilities of narration and allow for a posteriori quality control (Miller, 2009). The time of the therapeutic e-relationship is also less constrained than face-to-face interactions. It can either be contracted as chatting supposes a certain degree of immediacy, or dilated as the Web user can delay its responses. Overall, outreach intervention would require laying the foundation of a crisis e-clinic that would integrate counseling, motivational therapy, technological solutions, and Web-based communication.

## Discussion

**FIGURE 1:**
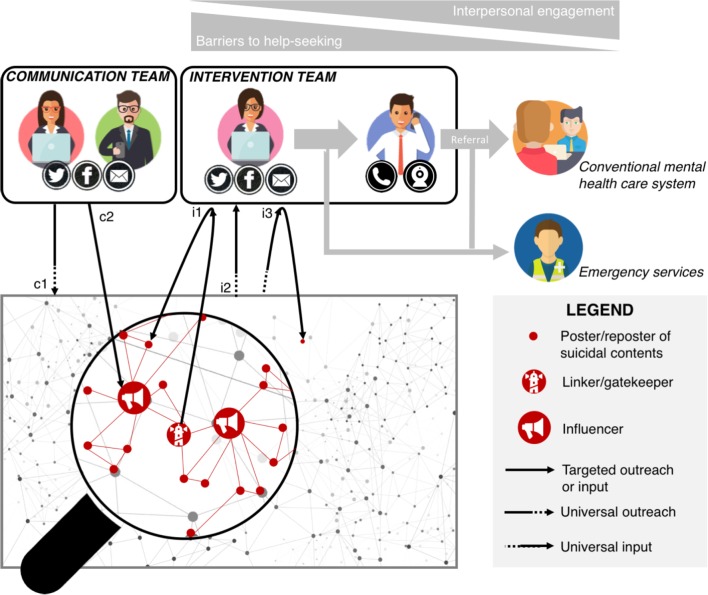
Proposal for an integrative e-prevention model based on social media. The graphical network stands for the generic functional and structural relationships between Web users, whatever the type of social media. The magnified red lines and dots represent the network of users who post suicidal content, as described in Section “The Web as a Gateway to Formal Mental Healthcare.” The e-prevention system has two components. The first operational team is in charge of the communication outreach strategy. Its function is to broadcast evidence-based prevention messages to promote help seeking. The communication plan includes universal messages disseminated indistinctly throughout the network (c1) and messages targeting suicidal Web users, possibly relayed by the network influencers (c2). The second operational team is composed of e-clinicians who conduct Web interventions and deliver referral services to distressed social media users. There are three ways for the intervention team to get informed of individuals who potentially need support (three input channels). The two first inputs are universal in that they can come from any point of the whole network: direct contact by suicidal individuals or relatives whose help-seeking process was enabled by the gateway function of the platform (i1); or detection of at-risk posters through data mining and machine learning procedures (i3). The last input is said to be targeted because it is known to be coming from the network of suicidal-posting users. It consists in reporting worrisome messages by network members, either ordinary users or linkers formally involved as gatekeepers (i2). In i2 and i3 cases, the intervention team reaches out to the identified individuals and queries their actual suicide risk and need for help. The intervention team has four main missions: (1) provide a first relief (counseling function); (2) activate and support the emergency services (dark blue pastille) whenever necessary (crisis function); (3) progressively increase the commitment of Web users in the help process (motivational function); and (4) refer Web users to the appropriate conventional healthcare services (yellow pastille). To carry out its missions, the intervention team can progressively switch from private text messaging (pink pastille) to interactive means requiring greater interpersonal commitment (such as vocal or videoconferencing – mauve pastille). Icons retrieved for www.flaticon.com.

The Internet has become an intrinsic component of our social landscape and daily life. As such, it cannot be ignored when dealing with suicide prevention. We believe that conditions are met to leverage the Internet to address one of the major public mental health issues, namely access to care. More specifically, we propose a model that uses social media as a transitional tool to initiate and reinforce the connection between ambivalent suicidal individuals and the formal healthcare system. The model, presented in **Figure 1**, coordinates the three pro-help-seeking functions presented in this paper, i.e., gateway, communication outreach, and intervention outreach. It relies on the synergy of two operational teams, both taking action via the main community, network and microblogging social media platforms. The Web communication team is composed of community managers and e-communicators, i.e., Web specialists able to design efficient communication campaigns tailored to the social media codes and constrains. They must prove a strong mastery of technological tools and specific skills in networking and social marketing. The Web intervention team consists of Web clinicians, either social workers, psychiatric nurses, psychologists, or psychiatrists. They should be specially trained in text-mediated counseling and crisis intervention, as well as in the use of social media technologies.

In practical terms, the model operates in two stages. The first stage consists in “phishing” suicidal Web users to create a first contact, either blindly (universal outreach) or after detection of worrisome posts (targeted outreach). The machine learning algorithms and the alerts of the gatekeepers allow the intervention team to directly approach the users who posted suicidal contents. Complementarily, the pro-Papageno campaign lead by the communication team gives an opportunity to incite mute suicidal Web users (also called “passive users”) to spontaneously contact the intervention team. The second stage begins as soon as the contact is established. Let us assume that the intervention team has intercepted a tweet stating “*Life is meaningless. Goodbye*,” and it has successfully created an interaction with its author via the Twitter private messaging service. The intervention team will then have to handle four complementary missions: (1) bring a first relief to the distressed Web user by providing active chat counseling; (2) evaluate the actual suicidal risk and mobilize the emergency services if necessary; (3) progressively increase the commitment of the Web user in the help process thanks to motivational support; and (4) refer the suicidal Web user as soon as possible to appropriate mental health services, possibly by scheduling the appointment for him/her. To achieve these goals, the modularity of the social media can be relevant and useful. The Web clinicians may prompt the patient’s therapeutic engagement by progressively proposing interfaces of growing inter-personal involvement, switching from text-based interactions (e-mails and chat), to online call, video-conferencing, and finally formal face-to-face meeting.

Importantly, the model we propose should not be considered an alternative to the traditional mental healthcare system, but rather as potential that increases its scope, helps to reach typically inaccessible populations, and reinforces the alliance with patients. This implies close collaboration between the operational Web teams and both the emergency services and mental health professionals. The Web community resources are also integrated as a key component of the system. As outlined in the literature, Web users by far anticipated the involvement of mental health professionals in dealing with suicidal contents on social media (Robinson et al., 2016a; Chan et al., 2017). Notably, peer-support and peer-surveillance initiatives were formally fostered by several platforms that created dedicated reporting systems for concerning posts (Facebook, 2015; Twitter, 2018). In our model, Web users – either ordinary network members or *linkers* involved as gatekeepers – could play a role in informing about worrisome posts. Such collaboration is anchored on the principle of mutual support: while the intervention team assists Web users in helping distressed peers, signalers of suicidal messages help to carry out outreach interventions.

We acknowledge that our model could crystallize sensitive ethical concerns such as how to keep a balance between the duty of care for presumed at-risk posters who did not formally ask for help and the principles of freedom of speech or privacy (Eggertson, 2015; Robinson et al., 2016a). More generally, online interventions are still at their infancy, and guidelines related to confidentiality (Robinson et al., 2016a), clinical safety (Luxton et al., 2012), and acceptability (Barak, 2007) issues must still be developed (Christensen et al., 2002).

The efficacy of our model remains to be tested. Due to the specific challenges that both Web interventions and complex public health actions pose, such an evaluation would require research innovation. For instance, methods remain to be developed to deal with pseudonyms rather than actual identities in the sampling and follow-up procedures (Lai et al., 2014). Furthermore, any attempts to establish causality assumptions between the online global strategy and its putative impact on the population would require a special effort to combine high-standard designs on both the individual and population levels, with a thoughtful choice of distal, proximal, and intermediary outcomes. In that regard, *infodemiology*, a branch of epidemiology that uses digital meta-data (e.g., Google queries, number of tweets) as proxies for “real life” indicators, could be relevantly exploited (Jashinsky et al., 2014; Sueki, 2015). Finally, the combination of qualitative and quantitative methods would help gain insight on the intrinsic socio-psychological process that underlies the impacts of the system (Lai et al., 2014).

If proved efficient, our model could formalize a trend reversal in prevention policies. Rather than simply attempting to counteract the adverse effect of social media, it would be about using them as an opportunity to increase prevention strategies. We believe this would result in general efficacy improvement, organizational optimization, and possibly cost reductions.

## Author Contributions

C-EN conceived the original idea, conducted the review of literature, set of conceptual basis of the model, and led the writing of the manuscript. PG, NP, MM, and MW substantially contributed to the article by identifying relevant references, providing conceptual inputs, and helping with the writing of the manuscript. MS and GV supervised the general process.

## Conflict of Interest Statement

The authors declare that the research was conducted in the absence of any commercial or financial relationships that could be construed as a potential conflict of interest.
